# Carotid arteries in cerebral small vessel disease and dementia

**DOI:** 10.1186/s40478-026-02250-w

**Published:** 2026-03-11

**Authors:** Erika Kitajima, Ashley Suwanda, Dan Jobson, Louise Allan, Kian Paydar, Gan Han, Kazuo Washida, Masafumi Ihara, Pazhanichamy Kalailingam, Yoshiki Hase, Siu Kwan Sze, Tuomo Polvikoski, Raj N. Kalaria

**Affiliations:** 1 Neurovascular Research Group, Translational and Clinical Research Institute, Campus for Ageing & Vitality, Newcastle University, NE4 5PL Newcastle Upon Tyne, UK; 2 University of Exeter Medical School, Exeter, UK; 3 Department of Neurology, National Cerebral and Cardiovascular Centre, Osaka, Japan; 4 Center for Genomic Medicine, Massachusetts General Hospital, Harvard Medical School, Boston, MA 02114 USA; 5 Faculty of Applied Health Sciences, Brock University, St. Catharines, ON L2S 3A1 Canada; 6 Translational and Clinical Research Institute, Campus for Ageing & Vitality, Newcastle University , Newcastle upon Tyne, NE4 5PL UK; 7 Himeji Dokkyo University, 7-2-1 Kamiohno, Himeji, 670-8524 Hyogo Japan

**Keywords:** Atherosclerosis, Carotid artery disease, Small vessel disease, Vascular dementia, White matter

## Abstract

Carotid artery disease (CAD) is a recognised cause of stroke. However, the relationships between CAD, cerebral small vessel disease (SVD) and dementia remain unclear. We hypothesised that CAD in older individuals contributes to cerebral SVD pathology by altering cerebral perfusion. We performed a clinicopathological study in patients from the Cognitive Function After Stroke (CogFAST) study and prospectively recruited patients with various dementia diagnoses and evidence of cerebral SVD. In addition to brain tissues, we collected postmortem samples of the internal carotid arteries (ICA) from these cohorts in the Newcastle Brain Tissue Resource. Standard neuropathological examination was performed for diagnosis and assignment of the cases per current diagnostic criteria for vascular and neurodegenerative dementias, which were assessed for the presence of vascular pathology including the degree of stenosis and sclerosis in vascular tissues. We evaluated a total of 159 ICA samples and brain tissues from all cases with evidence of SVD. Severity of ICA stenosis and sclerotic index correlated strongly with both clinical stroke and brain infarction (*P* < 0.001). More than 90% of the subjects had one subtype of ICA lesion in the order: intimal thickening > fibrocalcific > fibrous cap (thick) > fibrous cap (thin) > thrombus group with a strong inflammatory reaction in fibrocalcific atheromas. Regression analyses showed that ICA stenosis was positively correlated to both SVD pathology scores (*P* < 0.034) and the total number of vascular lesions (*P* < 0.001). ICA stenosis was also related to dementia caused by cerebrovascular disease (*P* < 0.001) and by mixed pathologies characterised by Alzheimer’s disease and SVD (*P* = 0.025). Severity of stenosis was related to subcortical and white matter (WM) vascular lesions within the anterior circulation. ICA stenosis and sclerosis were moreover correlated with the total intracranial artery pathology scores (*P* < 0.001). In the CogFAST group analysis, we observed that MMSE and CAMCOG scores were lower in subjects with moderate to severe stenosis scores compared to the mild stenosis group (*P* < 0.05). In these CogFAST cases, by far the majority of lesions in the WM were small in size (< 5 mm, range 72–91%) but not in the cortex or basal ganglia and thalamus. Linear regression analysis further indicated that there were greater numbers of these small lesions in the WM with increasing severity of ICA stenosis (*P* < 0.05). Our observations suggest carotid atherosclerosis promotes cerebral SVD types of change within the intracerebral arteries. It is conceivable that extracranial ICA pathology may influence perfusion and integrity of subcortical structures including the deep WM.

**Supplementary Information:**

The online version contains supplementary material available at 10.1186/s40478-026-02250-w.

## Introduction

Carotid artery disease (CAD) is estimated to cause 10–20% of all ischaemic strokes. Population based studies reported that the age-standardised incidence rates of ischaemic stroke subtypes due to large-artery atherosclerosis is approximately 15 per 100,000 persons [31]. This readily accords with the annual rate of first-ever and recurrent stroke attributed to extracranial internal carotid artery (ICA) as 13.4 (11.4–15.4) per 100,000 persons [11]. Carotid intima-medial thickness and carotid atherosclerotic plaques are also acknowledged as early and measurable risk factors for incident stroke and cerebrovascular events [23, 56, 57]. ICA stenosis classified as mild to moderate according to ECST or NASCET criteria is generally considered haemodynamically insignificant, and carotid endarterectomy (CEA) offers little to no clinical benefit in these cases. However, severe ICA stenosis is associated with a substantially increased risk of stroke, through two main mechanisms including haemodynamic impairment in case of significant stenosis and thromboembolism from an atherosclerotic plaque irrespective of the severity of stenosis. Severe ICA stenosis thereby warrants vascular intervention such as CEA [49], to reduce risk of stroke in both symptomatic and asymptomatic patients [3, 12, 18, 43].

CAD or carotid artery stenosis is also associated with markers of cerebral small vessel disease (SVD) such as white matter (WM) changes and lacunar infarcts [2, 30]. Again, SVD type of changes could result from haemodynamic as well as small thromboembolic originating from the carotid artery plaques. However, there is some evidence to suggest CAD could promote global hypoperfusion [33], cognitive impairment [51] and vascular dementia (VaD) [10, 46]. There are limited studies on the relationship between pathological CAD and intracranial brain vascular or parenchymal changes and dementia. In an attempt to fill gaps in the field, we assessed the spectrum of CAD in relation to cerebral SVD pathology in prospectively collected carotid arteries from two cohorts namely the Cognitive Function After Stroke (CogFAST) and Newcastle prospective dementia studies where we had clinical, neuroimaging and pathological evidence of cerebral SVD. We also attempted to address whether severity of stenosis is associated with dementia diagnosis particularly in the CogFAST cohort.

## Materials and methods

### Study design and subjects

Demographic, clinical and pathological findings in the subjects of the study are given in Table 1. Study subjects were participants of the Newcastle longitudinal prospective dementia series [20] and the CogFAST study. They had a clinical diagnosis of ischaemic stroke, vascular dementia, Alzheimer’s disease, mixed dementia (Mixed), dementia with Lewy bodies or Parkinson’s disease with dementia but all had some evidence of cerebral SVD. The Diagnostic and Statistical Manual of Mental Disorders, 4th Edition was used to further separate the post-stroke survivors into those with or without a clinical diagnosis of dementia in the CogFAST cohort [1]. Age-matched control subjects aged > 70 years were either part of previous prospective studies or from a pool of unrelated brain donations to the Newcastle Brain Tissue Resource (NBTR). They were included as ageing controls if they had not been diagnosed with cognitive impairment or any significant pathologies (Table 1) involving stroke, neurological or psychiatric illness. Additionally, subjects that did not show clinical evidence of overt stroke were utilised as neurological controls to comprise the ‘no stroke’ group.

Ethical approval and permissions for this study using donated human brains was granted by the Newcastle and North Tyneside 1 Research Ethics Committee and facilitated by the NBTR. Permission for use of brains for post-mortem research was also granted by consent from the participants themselves, next-of-kin or family member. Brain tissues were retained in and obtained from the NBTR.

**Table 1:** Demographic details of carotid artery cases in relation to clinical stroke, brain infarcts and dementia

Variable	Total No. of Cases	Clinical Stroke	No Stroke	Significance* (*P* value)
Case Number (*n*)	159 (100%)	104 (65.4%)	55 (34.6%)	-
Age (years)†	85.0 *±* 0.6	86.6 *±* 0.6	82.0 *±* 1.1	0.001
Age Range (min-max)	38 (63–101)	31 (68–99)	38 (63–101)	-
Sex: (Men %)	56%	50%	43%	0.104
Dementia (%)	61.1%	62.6%	59.4%	0.620
Brain Weight (g)	1228 *±* 11	1238 *±* 14	1210 *±* 21	0.442
Vascular Risk Factors (VRFs)††				
Hypertension (Systolic > 140 mm Hg) %	68.0%	62.2%	88.9%	< 0.01
Type 2 Diabetes %	15.1%	10.1%	33.3%%	< 0.01
Hypercholesterolemia %	18.3%	13.1%	37.0%	< 0.01
Mean No of VRFs (range)	1.6 (0–3)	1.6 (0–3)	1.6 (0–3)	> 0.05
Brain Infarction (%)	77.4%	96.2%	41.6%	<0.001
Neuropathological Findings‖				
NSP (control %)	11.3%	2.8%	27.3%	< 0.001
Cerebrovascular (%)	55.3%	80.0%	9.1%	< 0.001
Primary Neurodegenerative (%)	20.8%	4.8%	50.9%	< 0.001
Mixed Pathology (%)	12.6%	12.4%	12.7%	> 0.05
CAD Pathology‡				
Left ICA % External Diameter, mean *±* SEM cm	0.539 + 0.019	0.640 + 0.021	0.365 + 0.013	< 0.001
Right ICA % External Diameter, mean *±* SEM cm	0.537 + 0.018	0.644 + 0.020	0.365 + 0.012	< 0.001
Both ICA % External Diameter, mean *±* SEM cm	0.537 *±* 0.017	0.637 *±* 0.019	0.372 *±* 0.011	< 0.001
Left ICA Stenosis (%)	55.8 *±* 1.2	60.3 *±* 1.7	49.3 *±* 1.4	< 0.001
Right ICA Stenosis (%)	54.6 *±* 1.3	59.3 *±* 1.8	47.7 *±* 1.2	< 0.001
Both ICA Stenosis (%)	56.1 *±* 1.2	61.3 *±* 1.6	48.5 *±* 1.1	< 0.001
Left ICA Sclerosis (SI)	0.349 *±* 0.01	0.387 *±* 0.02	0.295 *±* 0.01	< 0.001
Right ICA Sclerosis (SI)	0.342 *±* 0.01	0.381 *±* 0.02	0.284 *±* 0.01	< 0.001
ICA Sclerosis (SI)	0.346 *±* 0.01	0.386 + 0.01	0.290 + 0.01	< 0.001
No. of ICA Lesions‡‡				
No. of apparent lesion (L/R)	15/17	4/7	11/10	> 0.05
Pathological Intimal Thickening (L/R)	48/45	24/28	24/17	> 0.05
Fibrocalcific (L/R)⁋	41/47	28/25	13/22	0.018
Fibrous Cap 1 (L/R)	37/37	24/22	13/15	> 0.05
Fibrous Cap 2 (thin) (L/R)	13/9	7/6	6/3	> 0.05
Other Occlusion e.g. thrombus (L/R)	2/1	2/1	0/0	> 0.05

Values are shown as mean ± SEM or % of total lesions unless otherwise specified. *Statistical significance between variables with or without clinical stroke was evaluated by ANOVA, post-hoc tests, independent t-test or Pearson Chi-square where applicable and variances were determined to be equal unless otherwise stated. †Mean ages (years + SEM) of men and women in the total sample were 83.4 ± 0.8 and 87.0 ± 0.8 (P = 0.001). There were no other significant differences between men and women for any of the variables including ICA stenosis/sclerosis, type of ICA, cerebral parenchymal pathology or vascular risk factors (VRFs) (P > 0.05). ††Values show percentage of individuals in each group reported to exhibit the relevant risk. Multiple regression analysis incorporating that whole sample indicated ICA stenosis and sclerosis were negatively correlated with hypertension, tested as a binary variable, (Model 1, r= -0.287, F9, df 1, P = 0.003 and r= -0.304, F10, df 1, P = 0.002) but no correlation with Type 2 diabetes (*P* = 0.545) or hypercholesterolemia (*P* = 0.954). We also found that having 1, 2 or 3 VRFs were not significantly correlated with the degrees of ICA stenosis or sclerosis (*P* > 0.05). For VRFs: available information is here limited to the three risks shown. Other variables involving vascular risks included ischaemic heart disease, obesity and body mass index, which were recorded in too few subjects to evaluate with meaningful results. ‖Neurodegenerative pathologies included Alzheimer’s disease, dementia with Lewy bodies, limbic age-related TDP-43 encephalopathy (LATE), Huntington’s disease, progressive supranuclear palsy, multiple system atrophy and mitochondrial disorder; any of which could be the cause of clinical dementia. ‡CAD pathology. ‡Number of ICA lesions in the left (L) and right (R) segments. Different lesion types were greater in the left ICA than in the right ICA in stroke patients (P = 0.01). We noted that the external diameters of the ICAs were significantly larger in stroke patients than those without stroke. In addition, the mean external diameter was correlated with the degree of stenosis (P < 0.01). ⁋Greater number of fibrocalcific lesions in the stroke cases. Abbreviations: CA, carotid artery; CAD, carotid artery disease; ICA, internal carotid artery; L, left; No., number; NSP, no significant pathology; R, right; SEM, standard error of the mean; SI, sclerotic index

### Post-mortem carotid arteries and brain tissues

During post-mortem examination, the neck region was dissected so that the left and right carotid arteries could be visually inspected. The bifurcation region on both sides was then detached by transverse cuts through the internal and external carotid arteries at least 2 cm above the bifurcation and by transverse cut through the common carotid artery at least 2 cm below the bifurcation. The resulting carotid bifurcation specimens were fixed for up to four weeks. After decalcification in EDTA solution, each of the three branches (common, internal and external) were sub-dissected by transverse cuts beginning from the exact bifurcation, so that four 3–4 mm long segments were generated. The resulting four samples (segments) represented together the first 16 mm above and below the bifurcation. Samples were then embedded providing corresponding sections for microscopy to show the bifurcation level: 4 mm, 8 mm, 12 mm and 16 mm above the bifurcation for the internal and external CA, and 4 mm, 8 mm, 12 mm and 16 mm below the bifurcation of the common CA. Individual samples of the right and left ICAs were subsequently taken at 4 mm distal to the level of the carotid bifurcation for each case assessed in the study.

Brains were sampled bilaterally and assessed in accordance with the Newcastle brain dissection protocol. Standardised protocols were used for microscopic and macroscopic pathology assessment [14, 24]. Briefly, macroscopic infarcts if present were recorded by visual inspection during dissection, and subsequently their presence was confirmed by microscopy. The size and total number of infarcts (designated as vascular lesions) in both hemispheres in the cortex, basal ganglia and thalamus, white matter, brainstem and cerebellum were recorded as follows: <5 mm, 5–15 mm, 16–30 mm, 31–50 mm and > 51 mm. Haematoxylin and eosin (H&E) stain was used for neuropathological assessment including vascular pathology scores to confirm SVD pathology. In addition to the total number, vascular lesions in the anterior and posterior circulation territories as well as cortical and subcortical regions were determined for each case.

### White matter pathology

All the cases assessed for WM pathology were initially screened for demyelination as described by Ihara et al. [24]. The degree of demyelination was taken into account to devise a four-point scale from 0 to 3 which was scored as the absence of significant changes (0), mild (1), moderate (2) and severe (3). A score of 0 meant there were no significant changes in myelination (i.e. lack of rarefaction) and the presence of or microinfarcts, perivascular spaces and haemosiderin [8, 20].

### Carotid and cerebral artery pathologies

Histopathological evaluation of ICA was conducted using standard tinctorial stains such as H&E. CAD was categorised using a previously established classification system [38] with additional adherence to recent observations on calcification of atherosclerotic plaques [53]. CAD was grouped into five subtypes consisting of intimal thickening, fibrocalcific, fibrous C1 (thick), fibrous C2 (thin) and other which included thrombi. CAD pathology was rated by two investigators (TMP and RNK) with > 90% agreement. Further cellular characterization of the artery vessel walls and atheromatous material was undertaken by immunostaining via standardised methods in 10 post-stroke cases with varied stenosis for α-smooth muscle actin (α-SMA), CD68-positive macrophages and isoAsp-Gly-Arg (isoDGR) degenerative protein modifications (1:200-1:1000 dilutions) [21, 27].

Carotid artery stenosis was categorized into mild, moderate and severe based on modifications of the ultrasound and angiographic methods used in the European Carotid Surgery Trial (ECST) and the North American Symptomatic Endarterectomy Trial [45, 50]. Given our previous histopathological study [19], we ascertained that moderate stenosis was in range 50–75% matching the ECST low moderate category, while the severe stenosis (> 75%) matched the combined ECST high moderate and severe categories. Following this paradigm we fit all our cases including cerebral arteries into three basic categories of mild (< 50%), moderate (50–75%) and severe (> 75%) stenosis (Supplementary Fig. 1).

The ICAs (per above), the circle of Willis, and basilar, anterior or middle cerebral arteries were examined for the degree of stenosis and assigned scores of 0 to 3 for none, mild, moderate and severe. Total intracerebral artery scores were calculated as an average of all the cerebral artery scores.

### Measurements of stenosis and sclerosis

For quantitative analysis, we scanned two to three 10$\:{\upmu\:}$m thick H&E-stained sections that were cut from paraffin-embedded transverse ICA blocks. Sections with the highest degree of stenosis were scanned using a photo scanner (EPSON Perfection V700, Seiko Epson Corporation, Suwa, Nagano, Japan) [19]. Total area of the external margin of the adventitia (S1) and luminal area at the interior margin of the intima (S2) were measured using Fiji software [52]. The % diameter stenosis was calculated using the following formula: % diameter stenosis = (S1-S2)/(S1) ×100. The % area stenosis of both right and left ICAs were calculated separately.

To determine the degree of sclerosis, the length from four different points across the artery were taken between the furthest exterior margin (S1) of the adventitia and the luminal area at its inner margin (S2). In preliminary experiments, we tested the use of this modified method by assessing larger vessels with diameters greater than 1 mm. The following formula was used to compute the sclerotic index = (S1-S2)/(S1). The measurement result from four points were averaged to calculate the final sclerotic index. Sclerotic indices were computed from both the left and right ICAs, and a mean value was then calculated. All data analyses were performed by three investigators (EK, AS, YH, RK) blinded to case identities. The inter-rater reliability between investigators was > 90% for determining the stenosis and sclerosis changes.

### Neurodegenerative pathology assessment

Gallyas and Bielschowsky’s silver impregnation and tau immunohistochemistry (AT8 for pTau at 1:1000 dilution) were used to assess neuritic plaques and neurofibrillary tangles for the ‘Consortium to Establish a Registry for Alzheimer’s Disease’ plaque score and ‘Braak and Braak’ neurofibrillary tangle staging. Pathological diagnosis of VaD was assigned, if there was clinical evidence of dementia using the Diagnostic and Statistical Manual of Mental Disorders, 4th Edition and relevant vascular pathology in the general absence of neurodegenerative pathology, that is, Braak staging I-IV. Severity of cerebral amyloid angiopathy (CAA) was assessed using a four-point scale as described previously [35]. α-synuclein was the pathological alteration in Lewy bodies dementia and Parkinson’s disease with dementia [20]. Total SVD pathology scores were determined out of a total of 20 according to Deramecourt et al. [8]. To assess glial cell responses, immunohistochemical staining was undertaken in sections from the frontal deep WM. Antibodies to glial fibrillary acidic protein (GFAP) and CD68 were used as respective markers of astrocytes and activated microglia or perivascular macrophages, with the percent area quantified as described previously [20, 21].

### Statistical analysis

Raw data were analysed using GraphPad Prism 10 software (Boston, MA, USA) and IBM SPSS Statistics Version 29.0.1.1 (Armonk, NY, USA). Standard descriptive statistics were used to describe the total and CogFAST samples. Unless otherwise stated, data are presented as mean$\:\pm\:$SEM. The Shapiro-Wilk test was used for normality testing. Multiple regression analysis was performed using ICA stenosis or sclerosis as the dependent variable with vascular risk factors such as hypertension, Type II diabetes and hypercholesterolemia as the independent variables and entered in the analysis as nominal (binary) variables. Bivariate relationships of key characteristics e.g. age, brain weight, degrees of stenosis, sclerosis, vascular pathology scores, number of vascular lesions, etc. were examined using correlation analysis (Pearson’s R), Student independent t tests, or analysis of variance (ANOVA) as appropriate. Post-hoc authentication included the Tukey and Bonferroni tests. The percentages of stenosis and sclerotic index were compared between stroke and non-stroke cases. The differences between variables to baseline characteristics were also compared using Pearson’s Chi-squared test. In all analyses, a value of *P* < 0.05 was considered as statistically significant.

## Results

### Clinical Stroke, brain infarction and carotid artery disease

This study was based on clinical and pathological observations in a total of 159 cases for which we had clinical information as well as pathological assessments of the carotid arteries (Table 1). Of these, 104 had clinical evidence of stroke with 5% of the cases showing no apparent brain infarction at postmortem (Table 1). Unless otherwise specified in the sub-analysis, subsequent analysis involved 140 cases where we had isolated carotid artery samples. We found that clinically defined stroke was associated with older age as was brain infarction on pathological examination (*P* = 0.001). There were no differences in sex, brain weights or presence of clinical dementia prior to death between the stroke and non-stroke groups (*P* > 0.05). The numbers of subjects with dementia in the stroke sample was 50% whereas these comprised 43% in the non-stroke sample (Table 1).

**Fig. 1:**
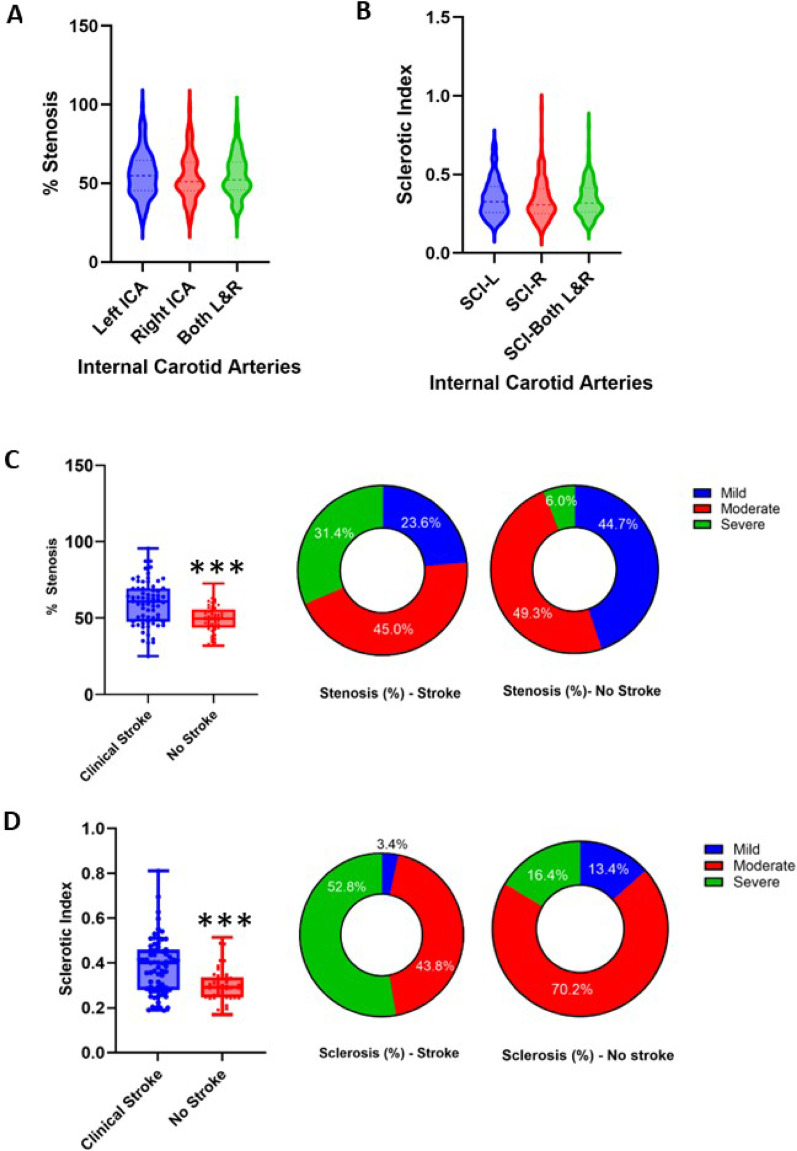
Carotid artery stenosis and sclerosis severity in clinical stroke. **A** and **B** Violin plots showing the distribution of % stenosis and sclerotic index (SCI) in left and right internal carotid arteries (ICAs) and the mean % in both ICAs. There were no differences in mean % stenosis or SCI between left and right arteries (*P* > 0.05). **C** Box plots and proportions of subjects with mild, moderate and severe stenosis among those with stroke and those without. **D** Box plots and proportions of subjects with mild, moderate and severe sclerosis among those with stroke and those without. *** *P* = 0.001 against the clinical stroke group. There were more cases with severe stenosis and sclerosis in subjects with clinical stroke (*P* = 0.001)

We first noted that neither the mean percent stenosis nor sclerosis was different between the left and right ICAs (Fig. 1A and B; Table 1). In subsequent analysis, we therefore averaged the percent values from left and right ICAs and used these as a single measure for each case. We found that the mean percent stenosis and sclerosis were greater in stroke subjects compared to non-stroke subjects (Table 1; Fig. 1C and D). In the total sample incorporating both stroke and non-stroke cases (*n* = 140), nearly 50% of the subjects exhibited moderate stenosis of the ICA with the rest as mild (32.7%) and severe (21.4%). The degree of stenosis and sclerosis differed significantly between the clinical stroke and non-stroke cases (Fig. 1C and D). More than 30% of the stroke cases showed severe stenosis compared to only 6% in non-stroke subjects. Similarly, severe sclerosis was also more common in stroke subjects; but remarkably 70% of the non-stroke samples exhibited moderate degrees of sclerosis in the ICA (Fig. 1D). Thus, stroke patients showed significantly worse ICA stenosis and sclerosis than those without stroke (Fig. 1), but moderate degrees of sclerosis were a common finding in the ICA irrespective of stroke pathology. ICA stenosis and sclerosis measures were strongly related to each other (*r* = 0.925, 95% CI 0.90–0.95, *P* < 0.001) but we also found correlations between clinical stroke and ICA stenosis and sclerosis, in a general linear model controlling for age (F16, df 1, *P* < 0.001 and F 15 df 1, *P* < 0.001, respectively). We found no other significant differences between men and women for any of the variables including ICA stenosis or sclerosis, type of ICA, cerebral parenchymal pathology and vascular risk factors (*P* > 0.05*)*. Chi square tests showed among the three vascular risk factors assessed; there were greater numbers of hypertensives found clinically in those without stroke compared to those with (Table 1). In further multiple regression analyses and modelling, we found ICA stenosis and sclerosis were surprisingly negatively correlated with hypertension, when tested as a binary variable, (Model 1, *P* = 0.003 and *P* = 0.002, respectively) but showed no correlation with Type 2 diabetes (Model 2, *P* = 0.545) or hypercholesterolemia (Model 3, *P* = 0.954). We also found that having 1, 2 or 3 vascular risk factors were not significantly correlated with the degrees of ICA stenosis or sclerosis (*P* > 0.05).

**Fig. 2:**
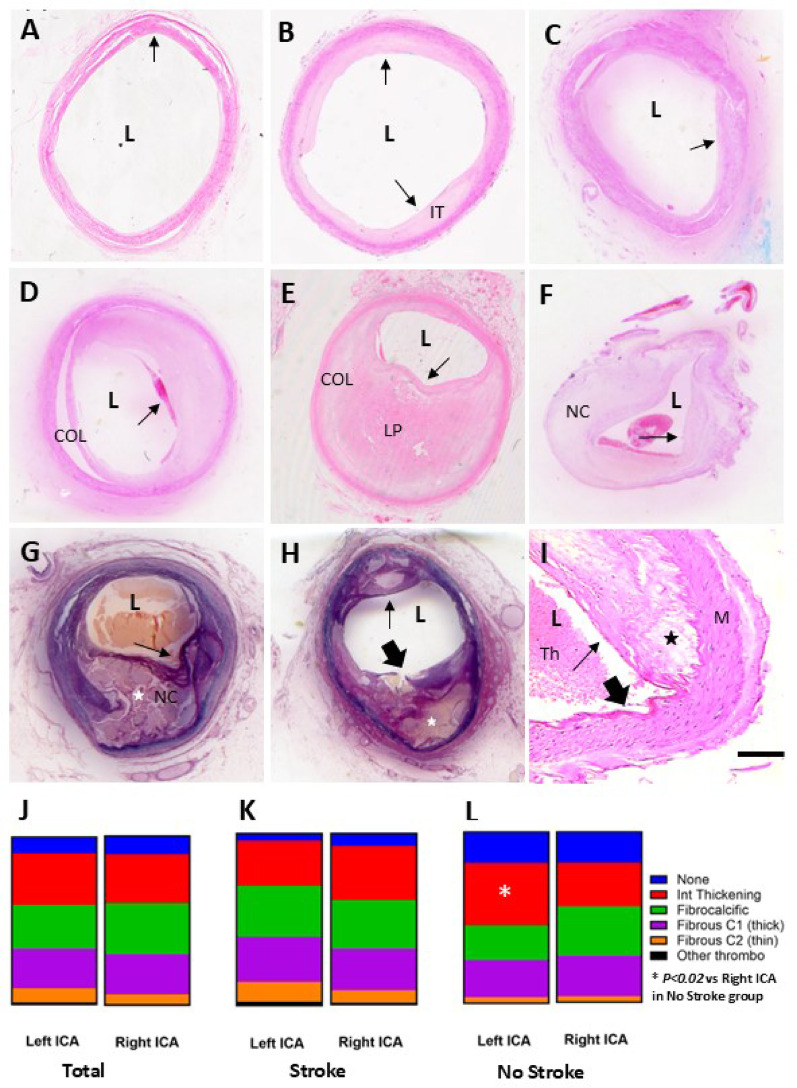
Types of carotid artery pathology in post-stroke patients. **A**-**I**, Representative images show five types of artery wall lesions within the internal carotid artery (ICA) causing various degrees of stenosis: intimal thickening (IT) (**B**-**C**), thin fibrous cap (**D-E**), thick fibrous cap (**F**), fibrocalcific (**G**-**H**) and other lesions (I) in post-stroke patients of 75–85 years of age. Thin arrows show the cap lesions where the thick arrow heads (H and I) indicate the ruptured cap (**H**) and intact intima (**I**). ICAs in G and H (stars) demonstrate examples of complicated lesions with a necrotic core (NC), calcified plaques and cholesterol clefts (star). I, cholesterol crystals (star) are often found in the vicinity of foam cells under a thick fibrous cap. Representative ICA sections stained with H&E (A-F, I) and Eosin Van Giessen (**G**-**H**). J-L, Proportions of lesion types in the left and right ICAs in the total sample (J), the clinical stroke (K) and no stroke (L) samples. Intimal thickening, fibrocalcific and thin fibrous cap lesions were the most common with generally no significant differences between left and right ICAs, except there were greater numbers of cases with intimal thickening in the left ICA compared to the right (* *P* < 0.02). Abbreviations: COL, collagen; H&E, haematoxylin and eosin; Int, intima; L, lumen; LP, lipid pool; M, media; thrombo, thrombosis; Th, thickening. Scale bar respectively represents 1 mm (**A**-**H**) and 200 μm (**I**)

**Fig. 3:**
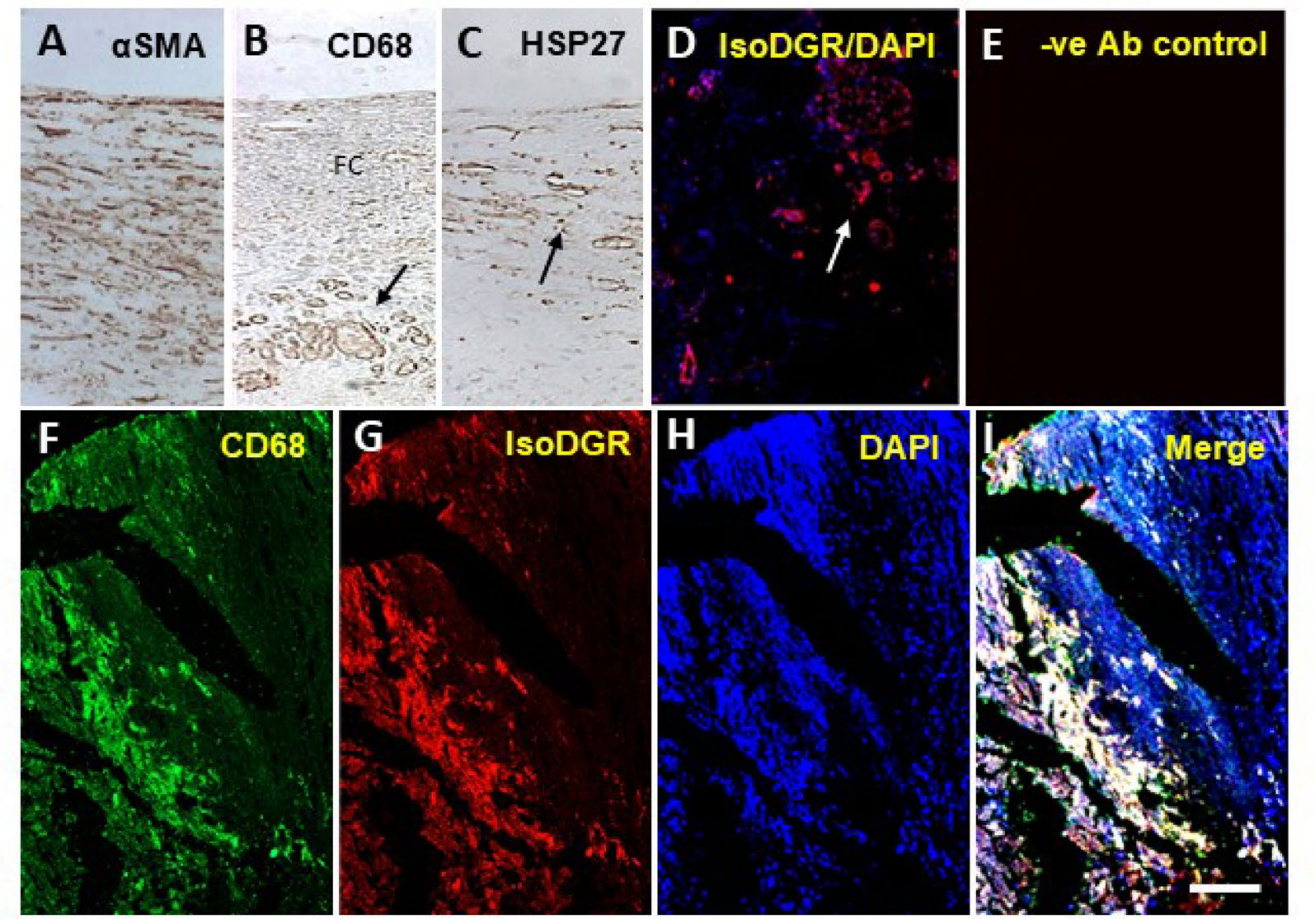
Internal carotid artery wall changes associated with atheromas and inflammation. **A**-**D** Immunohistochemical and immunofluorescence staining shows the distribution of α-SMA, CD68, HSP27 and isoDGR deposits relative to DAPI nuclei signal within the wall of carotid arteries. **A** Disbanded smooth muscle cells in the intima layer. macrophages, HSP27 and isoDGR (degenerative protein modifications, DPMs) in the walls of the vasa vasorum. **F**-**I** Images show high inflammatory activation of macrophages associated with DPMs in the internal carotid artery wall. Abbreviations: α-SMA; Ab, antibody; alpha-smooth muscle actin; DAPI, 4’,6-diamidino-2-phenylindole; isoDGR, isoAsp-Gly-Arg motifs; HSP27, heat shock protein 27. Scale bar represents 100 μm for **A-I**

We examined several segments of the common CA, the ICA and the external CA. As atherosclerosis was most frequent in the ICA, we undertook further analysis of at least three ICA segments at the same level. Macro- and microscopic examination of both the left and right ICA samples specified five different classic types of changes in the vessel walls (Figs. 2 and 3). More than 90% of the subjects had some type of ICA lesion in these age groups (Table 1). Light microscopy revealed that in general the atheromatous pathology in the ICA was not qualitatively different from that reported in coronary arteries or aortic arches [4, 38]. We found intimal thickening to be a common finding. The frequency in the order of the ICA subtypes in the total sample were intimal thickening > fibrocalcific > fibrous cap (thick) > fibrous cap (thin) > other group (Table 1; Fig. 2J-L). We also found the trend of marginally greater ICA pathology in the left than the right ICA, with only greater intimal thickening displaying significance in the no stroke group (*P* < 0.02). In addition, stroke subjects had greater proportions of pathological lesions in both left and right ICAs compared to those without stroke. However, there were substantial proportions of fibrocalcific and fibrous C1 (thick) lesions in both groups (Table 1) and across all confirmed brain pathologies including vascular, neurodegenerative and mixed (Supplementary Fig. 2). Ruptured atheromatous lesions were rare with a frequency of < 5% in the total sample suggesting there may be limited contribution by ICAs to thromboembolic strokes in the brain.

In order to identify specific changes within the intima and the atheromas in the vessel walls, we immunostained segments of the ICA for cellular inflammatory responses (Fig. 3). We found frequent disarray of α-SMA positive cells within the vessel wall matrix. Several cells were positive for heat shock protein 27, which we determined as typical foam cells. Numerous macrophages were also evident particularly in the medial layers in all the cases with fibrocalcific or fibrous cap lesions. We noted several vascular profiles depicting proliferation within the vasa vasorum. There was occasional evidence of haemorrhage within the atheromas possibly suggesting bleeding from weak vasa vasorum or other mechanisms (Fig. 2D; Supplementary Fig. 3). In addition, as we showed recently [26, 27] there was extensive accumulation of isoDGR-modified (deaminated) proteins [13] in the vessel walls overlapping with CD68-positive macrophages. This indicated an active and sustained inflammatory reaction ongoing within the ICA atheromas, potentially exacerbated by the accumulation of isoDGR-modified proteins [39, 47] (Fig. 3).

### Relationship between ICA pathology and cerebral SVD lesions

**Fig. 4:**
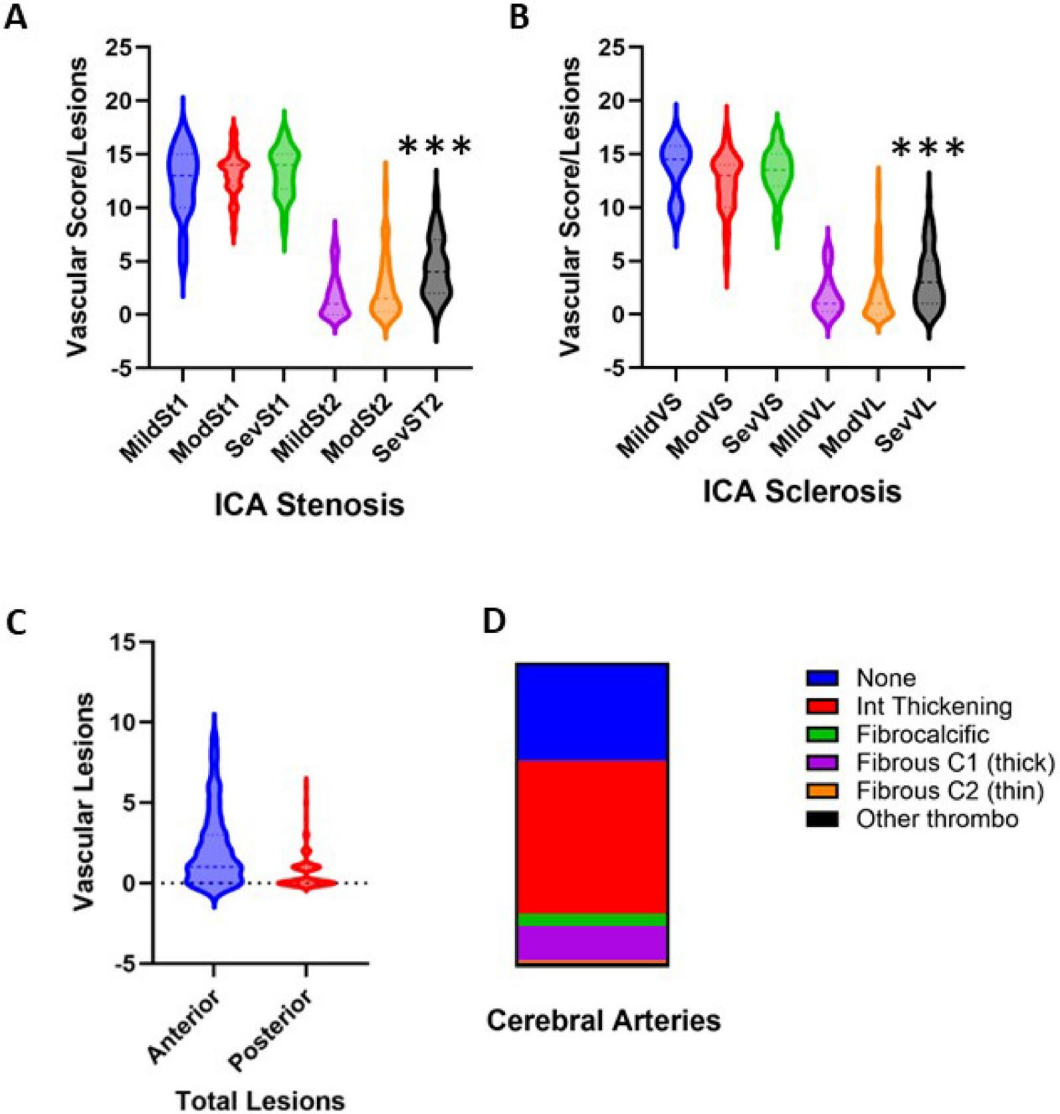
Brain SVD pathology and total number of lesions in relation to carotid artery pathology. A and B, Violin plots showing vascular SVD pathology scores and vascular lesions separated into mild, moderate and severe groups of CA pathology. *** *P* = 0.001 against mild (MildVL) or moderate (ModVL) in CA stenosis and CA sclerosis groups (**A**-**B**). **C** Violin plots showing vascular lesions associated with the anterior and posterior circulations. *** *P* = 0.001 against anterior circulation (C). **D** Proportions of lesion types in the whole sample of cerebral arteries. Intimal thickening was the most common type of arterial change. Abbreviations: CA, carotid artery; Mod, moderate; Sev, severe; St, stenosis; SVD, small vessel disease; VL, number of vascular lesions; VS, vascular small vessel disease pathology scores [8].

We next determined whether ICA stenosis influenced parenchymal pathology characterised by cerebral SVD (Supplementary Table 1). Linear regression analysis showed that ICA stenosis was positively related to both SVD pathology scores (*r* = 0.26, 95% CI 0.21–0.47, *P* < 0.034) and the total number of vascular lesions (*r* = 0.34, 95% CI 0.18–0.49, *P* < 0.001) (Supplementary Table 1). We further noted that subjects with both severe stenosis and sclerosis exhibited greater numbers of vascular lesions (Fig. 4A and B). We found subcortical rather than cortical vascular lesions to be related to severity of stenosis (Supplementary Table 1). Remarkably, WM scores (of vascular aetiology) were related to both ICA stenosis and sclerosis (Supplementary Table 1). These results suggest ICA pathology could have a role in altering the perfusion of the deep WM.

In terms of brain vascular pathology, we observed that cerebral vascular systems within the circle of Willis, basilar and smaller (anterior or middle) cerebral arteries characterised by any atheromatous disease or vessel wall lesions were all related to both ICA stenosis and sclerosis severity (Supplementary Table 1). After averaging scores for all cerebral artery pathology, we showed that the total intracranial artery scores were positively correlated with ICA stenosis and sclerosis (*r* = 0.42, 95% CI 0.26–0.56, *P* < 0.001) and (*r* = 0.37, 95% CI 0.20–0.51, *P* < 0.001, respectively) (Supplementary Table 1). There were greater numbers of vascular lesions in the anterior circulation compared to the posterior circulation (*P* < 0.001) (Fig. 4C; Supplementary Table 1). Our results indicated that CAD in the ICAs increased risk of atheromatous diseases in the cerebral arteries within the anterior circulation. It was also observed that intimal thickening was the most common lesion in cerebral vessels in this age group (Fig. 4D).

### Relationship between ICA pathology and dementia

**Fig. 5:**
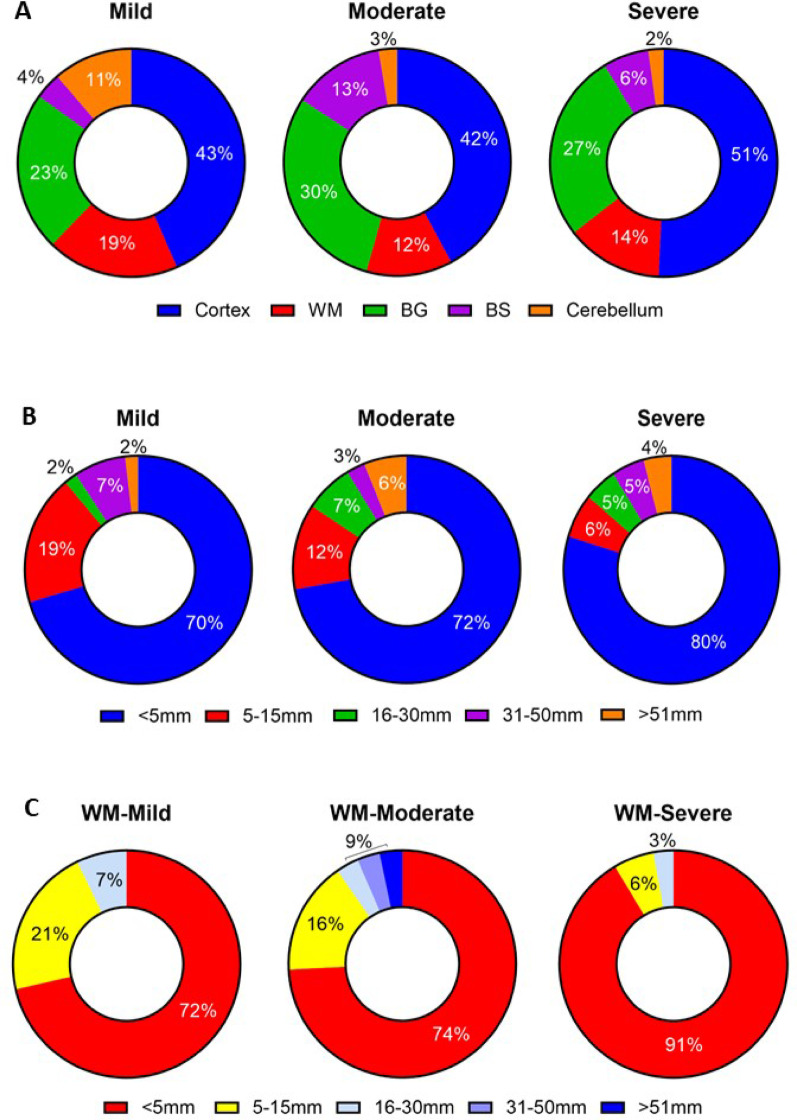
Pie charts showing location and size of brain infarcts in subjects with mild, moderate and severe internal carotid artery (ICA) stenosis. A, Distribution of infarcts in five different brain regions: cortex, white matter (WM), basal ganglia (BG) and thalamus, brainstem (BS) and cerebellum. Most infarcts were in the cerebral cortex with fewer numbers observed in the basal ganglia/thalamus, brainstem and WM across all mild, moderate or severe ICA stenosis subjects. Significantly more cerebellar infarcts were, however, found in the mild stenosis group (*P* < 0.05). B, Distribution of brain infarct size in patients with mild, moderate and severe ICA stenosis. Majority of the infarcts were <5 mm regardless of the degree of ICA stenosis. C, Size distribution of WM infarcts in subjects with mild, moderate and severe ICA stenosis. More than 70% of the infarcts in the WM in all three groups were <5 mm in size and those in subjects with severe ICA stenosis had proportionally more lesions of the smallest size e.g. 91%. Linear regression analysis showed severity of ICA stenosis was related to the smallest WM lesions (*r* = 0.42, 95% CI 0.27–0.56, F3.1, df 50, *P* < 0.048)

We further explored if the type of dementia was associated with the ICA pathological features in terms of either stenosis and/or sclerosis (Supplementary Tables 2 A and B). Regression analysis showed that ICA stenosis was positively related to both cases diagnosed as dementia caused by cerebrovascular disease (*P* < 0.001) and by mixed pathologies (*P* = 0.025) comprising cases with Alzheimer’s disease (Braak stage V and VI) and cerebrovascular lesions characterised by SVD type of pathology (Supplementary Table 2 A, Fig. 5B). Similarly, ICA sclerosis was also related to cerebrovascular disease (*P* < 0.001) and mixed dementia (*P* = 0.011) pathologies (Supplementary Table 2B).

### Carotid artery and SVD pathology in CogFAST

In an attempt to elucidate more specific substrates within stroke subjects, we assessed whether the severity of ICA stenosis, as mild, moderate or severe, was associated with a number of clinical and pathological variables specifically in the CogFAST study cases (Table 2). Of the 80 subjects we assessed, 23% exhibited mild stenosis, 38% had moderate stenosis and 39% had severe stenosis prior to death. As for the total sample: age, sex and brain weights did not differ significantly between the groups (Table 2). In terms of cognitive outcomes we found that at least 50% had a diagnosis of dementia in each group. While final Mini-Mental State Examination (MMSE) and Cambridge Cognition Examination (CAMCOG) cognitive scores tended to be reduced in the severe groups, we found that they were only significantly reduced when those with moderate to severe stenosis scores were combined and compared to the mild stenosis group (*P* < 0.05). CAMCOG scores in the severe group were lower than the mild group (*P* = 0.015).

**Table 2:** Demographic details of CogFAST cases in relation to severity of CAD and other pathologies

Variable	Mild	Moderate	Severe	Significance (*P* value)
Number of subjects (% of *n* = 80)	19 (23%)	30 (38%)	31 (39%)	-
Age (years), mean *±* SEM (range)	85.1 *±* 1.2 (76–95)	86.2 *±* 1.1 (71–98)	88.2 *±* 1.1(75–99)	0.156
Sex (male %)	53%	43%	61%	0.373
Brain Weight (g)	1247 *±* 40	1204 *±* 20	1271 *±* 27	0.179
Clinical Features				
Dementia (PSD) (%)†	52%	60%	58%	0.876
MMSE	24 *±* 1	21 *±* 2	20 *±* 2	0.250
CAMCOG	84 *±* 4	70 *±* 5	68 *±* 5	*< 0.05*
Hypertension (systolic > 140mmHg)	46%	76%	48%	0.233
Type 2 Diabetes	8%	5%	10%	0.771
Hypercholesterolemia	0%	14%	17%	0.290
No. of VRFs (range)	1 (0–3)	2 (0–3)	2 (0–3)	0.108
*APOE* Allele Frequencies				
* APOE ε4 (%)*	3.3%	5.0%	7.5%	> 0.05
* APOE ε3 (%)*	14.2%	26.7%	35.0%	> 0.05
* APOE ε2 (%)*	2.5%	1.7%	4.2%	> 0.05
Pathology Markers				
Left ICA % External Diameter, mean *±* SEM cm††	0.602 + 0.070	0.684 + 0.022	0.695 + 0.033	0.243
Right ICA % External Diameter, mean *±* SEM cm††	0.688 + 0.061	0.659 + 0.022	0.710 + 0.028	0.410
Both ICA % External Diameter, mean *±* SEM cm††	0.628 *±* 0.060	0.686 *±* 0.022	0.728 *±* 0.027	0.154
Left ICA % stenosis, mean *±* SEM‡	39.1 + 2.0	59.8 + 1.6	72.2 + 2.5	*< 0.001*
Right ICA % stenosis, mean *±* SEM‡	41.6 + 2.5	57.4 + 2.1	71.2 + 2.8	*< 0.001*
Both ICA % stenosis, mean *±* SEM‡	40.4 *±* 2.1	58.6 *±* 1.5	74.3 *±* 1.3	*< 0.001*
Left ICA % sclerosis, mean *±* SEM‡‡	0.194 + 0.00	0.296 + 0.02	0.469 + 0.02	*< 0.001*
Right ICA % sclerosis, mean *±* SEM‡‡	0.201 + 0.00	0.296 + 0.01	0.457 + 0.02	*< 0.001*
Both ICA % sclerosis, mean *±* SEM‡‡	0.247 *±* 0.013	0.370 *±* 0.014	0.482 *±* 0.021	*< 0.001*
Braak stage, mean (range)	2.8 (0–6)	3.0 (0–6)	2.8 (0–6)	> 0.05
CERAD score, mean (range)	1.0 (0–3)	1.2 (0–3)	1.1 (0–3)	> 0.05
α-synuclein score, mean (range)	0.0 (0)	0.7 (0–6)	1.7 (0–14)	> 0.05
CAA score, mean (range)	1.0 (0–3)	1.1 (0–3)	1.1 (0–3)	> 0.05
SVD Pathology score‖	12.6 *±* 0.8	13.0 *±* 0.4	13.3 *±* 0.4	0.589
Total cerebral vascular lesions	3.9 *±* 0.6	3.8 *±* 0.8	4.3 *±* 0.5	0.733
Intracranial artery score⁋	3.7 *±* 0.5	3.6 *±* 0.4	4.7 *±* 0.4	0.104
WM score	2.6 (1–3)	2.9 (2–3)	2.8 (1–3)	0.239
WM GFAP (% area)	0.82 *±* 0.1	0.91 *±* 0.1	1.30 *±* 0.15	*0.043*
WM CD68 (% area)⁋⁋	0.61 + 0.1	0.70 + 0.1	0.82 + 0.1	0.264

Values represent mean +SEM for the number of subjects shown. The range of severity scores in the circle of Willis, basilar artery and cerebral arteries was 1.1-1.7. There were no differences between mild, moderate and severe stenosed subjects (P>0.250). In the CogFAST cohort neither age nor sex was related to the severity of stenosis. †Prevalence of post-stroke dementia (PSD) defined by Diagnostic and Statistical Manual of Mental Disorders (DSM) IV, IVR or V criteria was not different in individuals with mild, moderate and severe CA stenosis or sclerosis in the CogFAST cohort (cf. Table 1). ††Represents the mean of left ICA and right CA, there were no differences between left and right ICA external diameters (P>0.05). ‡Severity of stenosis in both left and right CAs (ANOVA, P<0.001). ‡‡Severity of sclerosis in both left and right CAs (ANOVA, P<0.001). ‖SVD pathology and total vascular lesion scores were both correlated with ICA stenosis when analysed as continuous variables (see Supplementary Table 1). ⁋Intracranial artery score was correlated with both ICA stenosis and sclerosis when analysed as continuous variables (Supplementary Table 1). ⁋⁋ GFAP but not CD68 immunoreactivity was increased in the ICA severe stenosis group (ANOVA, n= 35, F3.3, df2, P=0.045). There were no significant differences between men and women for any of the cerebral SVD indices including total SVD pathology score, total number of cerebral vascular lesions or total intracranial artery scores (P>0.05). We noted only a trend in greater intracranial artery scores in women compared to men (t-test, 2.45 +0.24 and 3.24 +0.34 respectively, P=0.57). Abbreviations: ANOVA, analysis of variance; CA, carotid arteries; CAMCOG, Cambridge Cognition Examination; CERAD, Consortium to Establish a Registry for Alzheimer’s Disease; Braak, Braak NFT stage; CAA, cerebral amyloid angiopathy; ICA, internal carotid artery; PSD, post-stroke dementia; CBF, Cerebral blood flow; GFAP, glial fibrillary acidic protein; MMSE, Mini-Mental State Examination; PSD, post-stroke dementia; SEM, standard error of the mean; SVD, small vessel disease; VRF, vascular risk factor; WM, white matter.

There was no evidence for differences in frequencies or the number of vascular risk factors between the groups (Table 2). We also found no significant differences in the distribution of subjects with hypertension, Type II diabetes or hypercholesterolemia between the mild, moderate and severe ICA stenosis groups (Table 2). However, while there were no cases of hypercholesterolemia in the mild stenosis group, we noted that 14% and 17% of moderate and severe groups still exhibited this vascular risk factor. There were also no differences in the frequencies of *APOE ε2*,* ε3* or *ε4* alleles between the groups (*P* > 0.05).

Assessment of relevant pathological indices showed that mean values of both ICA stenosis and sclerosis were greater with increasing stenosis severity (*P* < 0.001). However, the distribution of vascular pathology or neurodegenerative disease markers was not different between the groups (Table 2). The prevalence of moderate-severe CAA pathology (approximately 24%) across all mild, moderate and severe ICA stenosis groups remained almost unchanged (Table 2). We also noted that WM pathology scores were almost similar in the mild, moderate and severe groups. However, ANOVA showed that frontal WM GFAP but not CD68 immunoreactivity was significantly increased in severe stenosis subjects relative to the mild stenosis group (*P* = 0.045).

In further analysis, we assessed the location and size of the lesions in the three groups (Fig. 5). These lesions were characterised as class II per the original Newcastle classification system [28]. Irrespective of the degree of ICA stenosis, we found that the greatest numbers of infarcts (range 42–51%) were in the cortex whereas the basal ganglia and thalamus contained the second highest numbers of infarcts (range 23–30%) (Fig. 5A). The cerebellum had the least numbers of lesions; although there were significantly more cerebellar infarcts in the mild stenosis compared to the moderate and severe stenosis groups (*P* < 0.05). In terms of the size most stroke lesions were small at < 5 mm, which encompassed 70% to 80% of the whole sample (Fig. 5B). There were no overall differences in the distribution of infarct size between the mild, moderate and severe stenosis groups (Fig. 5B). Further scrutiny of the data showed that by far most of the smallest infarcts (< 5 mm), comprising up to 91% in the WM were evident in the group with severe stenosis. Linear regression analysis indicated that increasing severity of ICA stenosis was related to the accretion of these smallest lesions in the WM (*P* < 0.048) (Fig. 5C and see legend).

## Discussion

Our clinicopathological analysis of a large sample of ICAs from both the CogFAST and prospective dementia cohorts confers several imperative findings and particularly throws light on the consequences of extracranial ICA stenosis on cerebral SVD. Focusing on the main findings, we found that (1) pathological evidence links increased ICA stenosis and sclerosis with SVD and WM lesions within clinically diagnosed stroke cases, (2) ICA atheromas were most commonly characterised by fibrocalcific lesions, (3) ICA stenosis (and sclerosis) was positively correlated with both SVD pathology scores and total number of vascular lesions, (4) severity of stenosis was associated with small infarcts in the subcortical structures including the WM rather than the cortex, (5) ICA stenosis and sclerosis severity were increased in dementia as explained by cerebrovascular or SVD pathology and by mixed pathologies (neurodegenerative and cerebrovascular) and (6) severe ICA stenosis was associated with multiple intracranial arteries by incorporating branches of the circle of Willis, anterior or middle cerebral arteries as well as the basilar artery.

Our first main observation on the severity of ICA pathology in stroke is perhaps not surprising as suggested by several clinical studies [29, 37]. We showed that moderate degree of stenosis and more importantly moderate to severe sclerosis was present in majority (~ 50%) of the cases assessed in this age group in this study. However, our findings also entail novelty in that fibrocalcific plaques in the elderly were the most common types of lesions in CAD. It has been suggested that atherosclerotic calcification initially occurs as microcalcifications [53] and results in larger dense calcification as evident in our cases. There also appears to be a predilection for the left ICA to accumulate distinct types of atheromatous lesions. Fibrocalcific plaques are relatively stable and may not necessarily contribute to ischaemic cerebrovascular events or SVD pathology. Previous clinical studies have indicated that high-risk plaques characterised by features such as echolucency, neovascularisation, lipid-rich necrotic cores and ulceration may increase ischaemic strokes [29], although high-risk plaques were not associated with incident stroke in patients with atrial fibrillation [44].

We found that while there were no qualitative differences between subtypes of atheromatous lesions described previously in the cardiovascular arteries [4], we found disarray of both αSMA positive cells and other features including foam cells, collagen and lipid clefts. This may not be surprising as atherosclerosis is a systemic inflammatory disease that associates with several acute cardiovascular complications triggered by atherosclerotic plaque rupture, which primarily manifests as stroke or myocardial infarction. We also showed that the inflammatory cellular response (demonstrated by CD68-positive macrophages) is concomitant with accumulation of degenerative protein modifications in extracellular matrix proteins such as fibronectin, laminin, and collagens [13, 15, 42]. The isoDGR-motif in the modified proteins is a ligand that specifically binds to integrin receptors, thus promoting immune cells recruitment and sustained inflammation, as detected by the isoDGR-specific antibody [39]. These observations are consistent with the varied transcriptomic and epigenomic characteristics of human carotid atherosclerotic plaques. A previous single-cell RNA and single-cell ATAC sequencing study identified 14 distinct cell populations including endothelial cells, smooth muscle cells, mast cells, B cells, myeloid cells and T cells in multiple cellular activation states and interconversions [7]. Thereby further emphasising the inflammatory cellular complexity of localised atherosclerotic plaque sites.

We showed that ICA stenosis and pathology were associated with a greater number of stroke lesions in the anterior compared to the posterior circulation. Our results accord with the population-based imaging studies showing that the carotid plaques are associated with infarction in the anterior circulation [23]. Our analysis also suggests that ICA stenosis influences the burden of cerebral SVD pathology, particularly in terms of vascular lesion number, collective SVD pathology scores as well as subcortical structures including the WM, which may be more vulnerable than other brain regions. We found that severity of both ICA stenosis and sclerosis was correlated with WM pathology and that mostly SVD type small lacunar lesions were present in the WM. These observations are consistent with clinical studies [30] and reinforce that not all WM changes evident, such as WM hyperintensities upon MRI of vascular origin, are solely explained by intracranial SVD. A systematic review followed by meta-analyses suggested that WM ipsilateral and contralateral to the CAD site is associated with changes in the apparent diffusion coefficient, fractional anisotropy and mean diffusivity values [2]. Consistent with our findings, this report indicates that CAD is also associated with quantifiable WM microstructural damage. We also suggest that the increased GFAP reactivity in the WM corroborates with our previous findings of astrocytic clasmatodendrosis in the frontal lobe deep WM of subjects in the CogFAST cohort, who exhibited greater volumes of WM hyperintensities and increasing degrees of demyelination [5].

Therefore, how could this hypoperfusion mechanism be explained? Since only a small proportion of CA atherosclerotic plaques i.e. other group we observed, could be classed as high-risk, we suggest haemodynamic impairment promoted by CA stenosis is likely the attributable mechanism involved in the cerebrovascular or cerebral SVD pathological changes as predominantly characterised by small infarcts (< 5 mm in size). However, we further suggest haemodynamic impairment possibly incorporating perturbed blood rheology is not the only contributor to SVD type of changes in the CogFAST and the total cohort, but it is not unlikely that microemboli from the cardiovascular system also play a role [37]. Thus, cerebral SVD events or deep watershed infarcts particularly in the WM may not only result from altered haemodynamics but also from microemboli alone through plaque inflammation as detected by fluorodeoxyglucose positron emission tomography imaging and the presence of transcranial Doppler microemboli signals [40]. With reference to the WM, it is also conceivable that CA stenosis induced haemodynamic changes in the proximal segments of the medullary and perforators lower the perfusion downstream affecting perfusion in the distal end arteries in WM. On the other hand in unstable plaques, it is plausible that macro-fragments from an atheroma may embolise or a thrombus may break off, leading to artery-to-artery embolism [32, 41] or there is cardiogenic microembolism due to atrial fibrillation [19, 36]. Irrespective, it needs to be emphasised that effects of antithrombotic agents on embolic signals should still serve as a marker for their efficacy in preventing stroke recurrence [16].

Previous work has suggested that cerebral SVD and WM infarction are also associated with increased risk of dementia [17, 25, 34, 55]. Although we did not observe a direct relationship between severity of stenosis and dementia per se possibly because dementia diagnosis is made as a nominal measure beyond a certain threshold. We did find cerebrovascular pathology was associated with dementia diagnosis in the total cohort and that both the MMSE and CAMCOG scores worsened with severity of ICA stenosis (moderate-severe group) in the CogFAST cohort. Furthermore, we did not find any sex or age-related differences in comparison to ICA stenosis or sclerosis in our sample, but subjects with stroke were older in age. To convey this finding in context, previous clinical studies showed that the prevalence of severe CA stenosis was ~ 3.1% (1.7% to 5.3%) in men aged 80 years or older and ~ 0.9% (0.3% to 2.4%) in women [6].

In addition, we provide evidence that severe ICA stenosis influences the vessel wall pathology or stenosis within multiple types of intracranial arteries that incorporate branches of the circle of Willis, anterior or middle cerebral arteries and the basilar artery. Thus, how might ICA stenosis and sclerosis affect cerebral vascular function? It is plausible that the carotid artery wall besides altering vascular haemodynamics may further affect endothelial cell function. In turn endothelium dysfunction may reduce vasomotor reactivity to impede normal flow leading to chronic or transient cerebral hypoperfusion particularly in the deeper distal structures including the WM. Relative to this reasoning, blood flow in the ICA without stenosis has been estimated to be 418 ml/min but with mild, moderate, and severe stenosis, it was calculated respectively to be 309 ml/min, 229 ml/min, and 117 ml/min [54]. Similarly, another study showed that with the increase in the severity of ICA stenosis, the ipsilateral blood flow reduced to 197 ml/min when stenosis was 50–69% and to 130 ml/min i.e. to 25% of normal flow with severe (70–99%) stenosis. Furthermore, the blood-brain barrier may also be compromised in the chronic hypoperfusion and hypoxic state [9, 48, 58]. We propose that intimal thickening within the intracranial (and intracerebral) arteries occurring as a function of age in tandem with changes in the haemodynamics and perfusion possibly exert progressive propagation effects along the arterial branching. We also propose that elucidation of left and right hemispheric vascular lesion burden separately in a larger cohort may enable to precisely determine the haemodynamic alteration mechanisms.

While our report comprises several strengths including pathology associated with ICA stenosis and its relationship with SVD pathology and declining cognition, there are several limitations of the study. First, we would need a larger unbiased sample size for the autopsy cohort to definitively determine the precise effect of ICA stenosis on cognitive function measures. Such an unbiased or random large sample would only be achieved in fit for purpose longitudinal studies involving collection of carotid arteries and determining comprehensive neuropathological evaluation in all collected cases irrespective of the pathological diagnosis. A larger sample with accompanying clinical records on vascular risk factors would also allow clearer understanding whether certain vascular risks are particularly associated with ICA stenosis and parenchymal pathology. In this context, it is difficult to explain why hypertension was negatively associated with ICA stenosis and sclerosis but studies have suggested variable relationships between carotid artery stenosis and vascular risk factors [22]. While our data on the presence or absence of hypertension above systolic 140 mmHg could be limited in detecting anticipated relationships between hypertension and ICA stenosis, it is also plausible larger numbers of cases, particularly with severe stenosis would have revealed a different outcome. Our study is also limited in that it remains unclear whether ICA stenosis and hence the resulting hypoperfusion influences the deposition of cerebral neurodegenerative pathologies in earlier stages of dementia. Another limitation would be the use of medications including antiplatelet agents, anticoagulants, statins and antihypertensives. These were not fully accounted for in the present analysis because the information on hand was not complete. In this respect, a potential future direction would be to investigate, particularly among individuals with severe ICA stenosis, whether these medications influence stroke risk, WM pathology, intimal thickening, fibrocalcific changes or fibrous cap characteristics.

In summary, we provide evidence that severity of ICA stenosis may alter the accumulation of cerebral SVD pathology and is associated with cognition. Moreover, subcortical structures including the WM may be more targeted than other brain regions. We also show that ICAs in the elderly are characterised by various subtypes of lesions among which intimal thickening and fibrocalcific lesions are common with significant presence of inflammation. We also suggest extracranial stenosis most proximal to the cerebrum likely affects vessel wall health in more distal segments of the cerebral microvasculature.

## Supplementary Information

Below is the link to the electronic supplementary material.

Supplementary Material 1. Supplementary Table 1: †Results show linear correlation analysis values for n = 137, which accounts for 156 pairs. Values represent Pearson correlations calculated assuming equal variances and significant correlations (P < 0.05) between the variables are displayed in bold. Sex was not related to carotid artery stenosis or sclerosis (Chi square test, P > 0.05). Yet there was a linear relationship between ICA stenosis and ICA sclerosis (Pearson r = 0.925, P < 0.001). ‡Dementia was defined by DSM IV, IVR or V criteria and was not associated with the severity of ICA stenosis or sclerosis (cf. Table 1). Abbreviations: DSM, Diagnostic and Statistical Manual of Mental Disorders; ICA, internal carotid arteries; SVD, small vessel disease; WM, white matter. Supplementary Table 2: Results of ANOVA showing the correlation between the degree of ICA sclerosis (A) or ICA sclerosis (B) and type of dementia pathology at diagnosis. Significant relationships (P<0.05) are shown in bold. *Compared to NSP; Tukey’s honestly significant difference and Dunnett’s post-hoc tests. †NSP, No significant pathology. ††Primary neurodegenerative disease included mainly AD, DLB or PD. ‡Mixed included AD + VaD or AD + DLB +VaD or PSP + VaD. Abbreviations: AD, Alzheimer’s disease; ANOVA, one-way analysis of variance; CI, confidence interval; DLB, dementia with Lewy bodies; ICA, internal carotid arteries; N/A, not applicable; N, number; PD, Parkinson’s disease; PSP, progressive supranuclear palsy; SE, standard error; VaD, vascular dementia.

Supplementary Material 2. Supplementary Figure 1: The diagram shows how carotid artery stenosis was categorised and determined into mild, moderate and severe forms based on modifications of the ultrasound and angiographic methods used in the European Carotid Surgery Trial (ECST) and the North American Symptomatic Endarterectomy Trial [45, 50]. Supplementary Figure 2: Graphs show the proportions of fibrocalcific and fibrous C1 (thick) lesions across all confirmed brain pathologies including none, vascular (Vasc), neurodegenerative (NeuroP) and mixed types. Abbreviations: int, intima; LICA, left internal carotid arteries; RICA, right internal carotid arteries; thrombo, thrombosis.

Supplementary Material 3. Supplementary Figure 3: Internal carotid artery wall changes within atheromas involving microhaemorrhages and inflammatory responses. A-D, Various sizes of bleeds within atheromas in internal carotid arteries with 50-75% stenosis (arrows). B, is an image at higher power of the area identified by the thick arrowhead in A. Inflammatory cells are also visibly observable (black arrowheads). EG, Different sizes of vessel profiles can be seen within the vasa vasorum (arrows; cf. Figure 2A), with some associated with bleeds (F). Inflammatory cell infiltrates are also evident (black arrowheads). A-D, images from two segments of the internal carotid artery at different levels from a 92-year-old man with post-stroke dementia. E-I, internal carotid arteries from a 92-year-old woman with vascular dementia (E-F) and 94-year-old with post stroke dementia (G-I). Abbreviations: Adv, adventitia; L, lumen. Scale bar respectively represents 500μm (A, C, D) and 200μm (B, E-I).

Supplementary Tables 1 and 2

Supplementary material 2 - supplementary Figures 1 and 2

Supplementary Material 3 - Supplementary Figure 3

## Data Availability

The data that support the findings of this study are available on request from the corresponding author. The data are not publicly available due to case privacy or ethical restrictions.
