# Supplementary material for: Carotid arteries in cerebral small vessel disease and dementia
Source: Acta Neuropathol Commun. 2026 Mar 11;14:94. doi: 10.1186/s40478-026-02250-w (PMC13088681; doi:10.1186/s40478-026-02250-w)
Supplement: Supplementary file 1 — Supplementary Material 1. Supplementary Table 1: †Results show linear correlation analysis values for n = 137, which accounts for 156 pairs. Values represent Pearson correlations calculated assuming equal variances and significant correlations (P < 0.05) between the variables are displayed in bold. Sex was not related to carotid artery stenosis or sclerosis (Chi square test, P > 0.05). Yet there was a linear relationship between ICA stenosis and ICA sclerosis (Pearson r = 0.925, P < 0.001). ‡Dementia was defined by DSM IV, IVR or V criteria and was not associated with the severity of ICA stenosis or sclerosis (cf. Table 1). Abbreviations: DSM, Diagnostic and Statistical Manual of Mental Disorders; ICA, internal carotid arteries; SVD, small vessel disease; WM, white matter. Supplementary Table 2: Results of ANOVA showing the correlation between the degree of ICA sclerosis (A) or ICA sclerosis (B) and type of dementia pathology at diagnosis. Significant relationships (P<0.05) are shown in bold. *Compared to NSP; Tukey’s honestly significant difference and Dunnett’s post-hoc tests. †NSP, No significant pathology. ††Primary neurodegenerative disease included mainly AD, DLB or PD. ‡Mixed included AD + VaD or AD + DLB +VaD or PSP + VaD. Abbreviations: AD, Alzheimer’s disease; ANOVA, one-way analysis of variance; CI, confidence interval; DLB, dementia with Lewy bodies; ICA, internal carotid arteries; N/A, not applicable; N, number; PD, Parkinson’s disease; PSP, progressive supranuclear palsy; SE, standard error; VaD, vascular dementia. [file 40478_2026_2250_MOESM1_ESM.docx]

**Supplementary Tables 1 and 2**

**Carotid Arteries in Cerebral Small Vessel Disease and Dementia**

Erika Kitajima,^1^ Ashley Suwanda,^1^ Dan Jobson,^1^ Louise Allan,^2^ Kian Paydar,^1^ Gan Han,^1^ Kauzo Washida,^3^ Masafumi Ihara,^3^ Pazhanichamy Kalailingam,^4^ Yoshiki Hase,^1^ Newman SK Sze,^5^ Tuomo Polvikoski,^1^ and Raj N. Kalaria^1*^

**Supplementary Table 1**

**Correlations between ICA stenosis or sclerosis with various cerebral pathologies**

| **Variable** | **ICA Stenosis†** | *P value* | **ICA Sclerosis†** | *P value* |
| --- | --- | --- | --- | --- |
| Age (yr) | 0.165 | 0.54 | 0.140 | 0.105 |
| Brain Weight (g) | 0.034 | 0.713 | 0.027 | 0.769 |
| **Parenchymal Pathology** |  | | | |
| Total SVD Pathology score | **0.258** | *0.034* | 0.182 | 0.142 |
| Total Vascular Lesions | **0.341** | *0.001* | **0.240** | *0.007* |
| Cortical Lesions | 0.073 | 0.491 | 0.054 | 0.615 |
| Subcortical Lesions | **0.246** | *0.019* | 0.136 | 0.205 |
| WM score | **0.485** | *0.001* | **0.423** | *0.001* |
| **Cerebral Circulation** |  | | | |
| Anterior Circulation | **0.339** | 0.001 | **0.239** | 0.010 |
| Posterior  Circulation | 0.129 | 0.164 | 0.064 | 0.495 |
| **Vessel Pathology** |  | | | |
| Circle of Willis | **0.337** | *0.001* | **0.314** | *0.001* |
| Basilar Artery | **0.420** | *0.001* | **0.348** | *0.001* |
| Cerebral Arteries | **0.387** | *0.001* | **0.349** | *0.001* |
| Total Intracranial Artery score | **0.420** | *0.001* | **0.365** | *0.001* |

†Results show linear correlation analysis values for *n*=137, which accounts for 156 pairs. Values represent Pearson correlations calculated assuming equal variances and significant correlations (*P*<0.05) between the variables are displayed in bold. Sex was not related to carotid artery stenosis or sclerosis (Chi square test, *P*>0.05). Yet there was a linear relationship between ICA stenosis and ICA sclerosis (Pearson *r*=0.925, *P*<0.001). ‡Dementia was defined by DSM IV, IVR or V criteria and was not associated with the severity of ICA stenosis or sclerosis (cf. Table 1). Abbreviations: DSM, Diagnostic and Statistical Manual of Mental Disorders; ICA, internal carotid arteries; SVD, small vessel disease; WM, white matter.

**Supplementary Table 2**

1. **Relationship between ICA stenosis and pathological diagnosis of dementia**

| Predominant type of pathology associated with dementia | ***N*** | **Mean** | **SE** | **95% CI for Mean** | | ***P* value*** |
| --- | --- | --- | --- | --- | --- | --- |
|  |  |  |  | Lower Bound | Upper Bound |  |
| NSP† | 18 | 46.5% | 2.7% | 40.9% | 52.3% | N/A |
| **Cerebrovascular disease** | **68** | **60.3%** | **1.8%** | **56.8%** | **63.8%** | **<0.001** |
| Primary Neurodegenerative disease†† | 33 | 50.9% | 1.5% | 47.9% | 53.9% | 0.643 |
| **Mixed**‡ | **17** | **58.8%** | **3.0%** | **52.4%** | **65.2%** | **0.025** |
| **Total** | **136** | **56.0%** | **1.2%** | **53.7%** | **58.3%** | **<0.001** |

**B) Relationship between ICA sclerosis and pathological diagnosis of dementia**

| Predominant type of pathology associated with dementia | ***N*** | **Mean** | **SE** | **95% CI for Mean** | | ***P* value*** |
| --- | --- | --- | --- | --- | --- | --- |
|  |  |  |  | Lower Bound | Upper Bound |  |
| NSP† | 18 | 0.267 | 0.02 | 0.230 | 0.304 | N/A |
| **Cerebrovascular disease** | **67** | **0.379** | **0.02** | **0.349** | **0.410** | **<0.001** |
| Primary Neurodegenerative disease†† | 33 | 0.305 | 0.01 | 0.283 | 0.328 | 0.598 |
| **Mixed**‡ | **18** | **0.377** | **0.03** | **0.323** | **0.430** | **0.011** |
| **Total** | **136** | **0.346** | **0.01** | **0.327** | **0.365** | **<0.001** |

Results of ANOVA showing the correlation between the degree of ICA sclerosis (A) or ICA sclerosis (B) and type of dementia pathology at diagnosis. Significant relationships (*P*<0.05) are shown in bold. *Compared to NSP; Tukey’s honestly significant difference and Dunnett’s post-hoc tests. †NSP, No significant pathology. ††Primary neurodegenerative disease included mainly AD, DLB or PD. ‡Mixed included AD + VaD or AD + DLB +VaD or PSP + VaD. Abbreviations: AD, Alzheimer’s disease; ANOVA, one-way analysis of variance; CI, confidence interval; DLB, dementia with Lewy bodies; ICA, internal carotid arteries; N/A, not applicable; *N*, number; PD, Parkinson’s disease; PSP, progressive supranuclear palsy; SE, standard error; VaD, vascular dementia.

**Supplementary Figure Legends**

**Supplementary Figure 1:** The diagram shows how carotid artery stenosis was categorised and determined into mild, moderate and severe forms based on modiﬁcations of the ultrasound and angiographic methods used in the European Carotid Surgery Trial (ECST) and the North American Symptomatic Endarterectomy Trial [43, 47].

**Supplementary Figure 2:** Graphs show the proportions of fibrocalcific and fibrous C1 (thick) lesions across all confirmed brain pathologies including none, vascular (Vasc), neurodegenerative (NeuroP) and mixed types. Abbreviations: int, intima; LICA, left internal carotid arteries; RICA, right internal carotid arteries; thrombo, thrombosis.

**Supplementary Figure 3:** Internal carotid artery wall changes within atheromas involving microhaemorrhages and inflammatory responses. A-D, Various sizes of bleeds within atheromas in internal carotid arteries with 50-75% stenosis (arrows). B, is an image at higher power of the area identified by the thick arrowhead in A. Inflammatory cells are also visibly observable (black arrowheads). E-G, Different sizes of vessel profiles can be seen within the vasa vasorum (arrows; cf. Figure 2A), with some associated with bleeds (F). Inflammatory cell infiltrates are also evident (black arrowheads). A-D, images from two segments of the internal carotid artery at different levels from a 92-year-old man with post-stroke dementia. E-I, internal carotid arteries from a 92-year-old woman with vascular dementia (E-F) and 94-year-old with post stroke dementia (G-I). Abbreviations: Adv, adventitia; L, lumen. Scale bar respectively represents 500μm (A, C, D) and 200µm (B, E-I).
