# Supplementary figures and images for: Carotid arteries in cerebral small vessel disease and dementia
Source: Acta Neuropathol Commun. 2026 Mar 11;14:94. doi: 10.1186/s40478-026-02250-w (PMC13088681; doi:10.1186/s40478-026-02250-w)

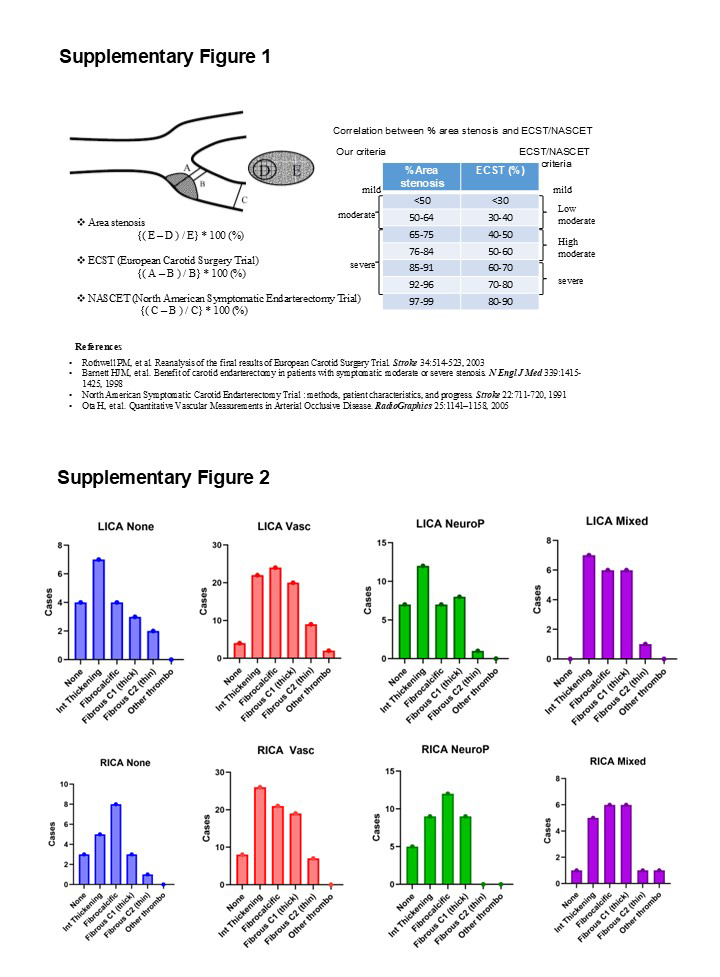

Supplement: Supplementary file 2 — Supplementary Material 2. Supplementary Figure 1: The diagram shows how carotid artery stenosis was categorised and determined into mild, moderate and severe forms based on modifications of the ultrasound and angiographic methods used in the European Carotid Surgery Trial (ECST) and the North American Symptomatic Endarterectomy Trial [45, 50]. Supplementary Figure 2: Graphs show the proportions of fibrocalcific and fibrous C1 (thick) lesions across all confirmed brain pathologies including none, vascular (Vasc), neurodegenerative (NeuroP) and mixed types. Abbreviations: int, intima; LICA, left internal carotid arteries; RICA, right internal carotid arteries; thrombo, thrombosis. [file 40478_2026_2250_MOESM2_ESM.tif]

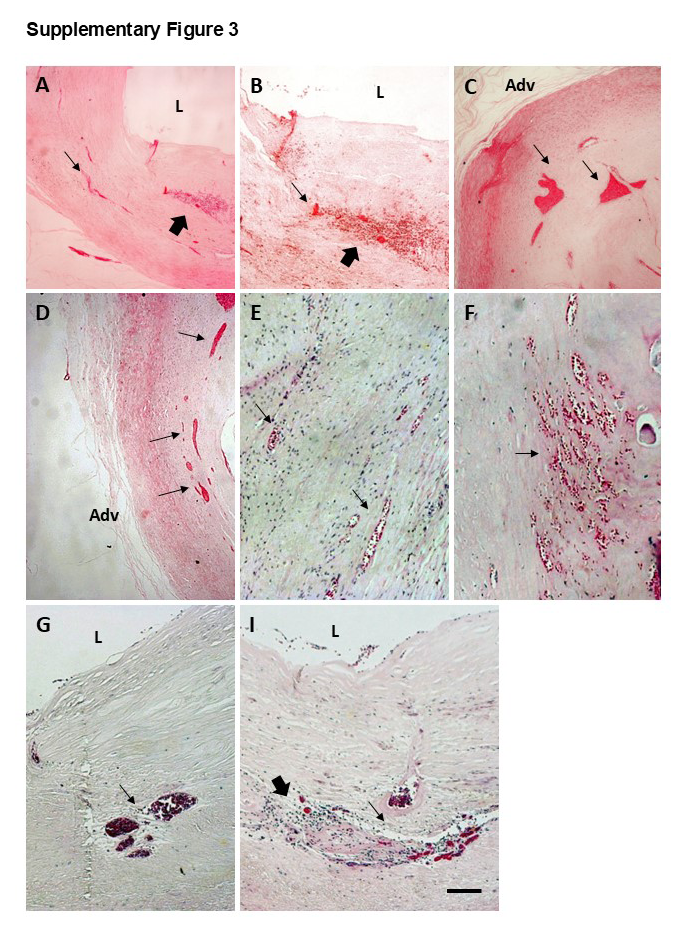

Supplement: Supplementary file 3 — Supplementary Material 3. Supplementary Figure 3: Internal carotid artery wall changes within atheromas involving microhaemorrhages and inflammatory responses. A-D, Various sizes of bleeds within atheromas in internal carotid arteries with 50-75% stenosis (arrows). B, is an image at higher power of the area identified by the thick arrowhead in A. Inflammatory cells are also visibly observable (black arrowheads). EG, Different sizes of vessel profiles can be seen within the vasa vasorum (arrows; cf. Figure 2A), with some associated with bleeds (F). Inflammatory cell infiltrates are also evident (black arrowheads). A-D, images from two segments of the internal carotid artery at different levels from a 92-year-old man with post-stroke dementia. E-I, internal carotid arteries from a 92-year-old woman with vascular dementia (E-F) and 94-year-old with post stroke dementia (G-I). Abbreviations: Adv, adventitia; L, lumen. Scale bar respectively represents 500μm (A, C, D) and 200μm (B, E-I). [file 40478_2026_2250_MOESM3_ESM.tif]
